# Quantifying sociodemographic heterogeneities in the distribution of *Aedes aegypti* among California households

**DOI:** 10.1371/journal.pntd.0008408

**Published:** 2020-07-21

**Authors:** Marisa A. P. Donnelly, Susanne Kluh, Robert E. Snyder, Christopher M. Barker

**Affiliations:** 1 Department of Pathology, Microbiology, and Immunology, University of California Davis, Davis, California, United States of America; 2 Greater Los Angeles County Vector Control District, Santa Fe Springs, California, United States of America; 3 Vector-borne Disease Section, Division of Communicable Disease Control, California Department of Public Health, Sacramento, California, United States of America; Australian Red Cross Lifelood, AUSTRALIA

## Abstract

The spread of *Aedes aegypti* in California and other regions of the U.S. has increased the need to understand the potential for local chains of *Ae*. *aegypti-*borne virus transmission, particularly in arid regions where the ecology of these mosquitoes is less understood. For public health and vector control programs, it is helpful to know whether variation in risk of local transmission can be attributed to socio-demographic factors that could help to target surveillance and control programs. Socio-demographic factors have been shown to influence transmission risk of dengue virus outside the U.S. by modifying biting rates and vector abundance. In regions of the U.S. where *Ae*. *aegypti* have recently invaded and where residential areas are structured differently than those in the tropics where *Ae*. *aegypti* are endemic, it is unclear how socio-demographic factors modify the abundance of *Ae*. *aegypti* populations. Understanding heterogeneities among households in *Ae*. *aegypti* abundance will provide a better understanding of local vectorial capacity and is an important component of understanding risk of local *Ae*. *aegypti-*borne virus transmission. We conducted a cross-sectional study in Los Angeles County, California during summer 2017 to understand the causes of variation in relative abundance of *Ae*. *aegypti* among households. We surveyed 161 houses, representing a wide range of incomes. Surveys consisted of systematic adult mosquito collections, inspections of households and properties, and administration of a questionnaire in English or Spanish. Adult *Ae*. *aegypti* were detected at 72% of households overall and were found indoors at 12% of households. An average of 3.1 *Ae*. *aegypti* were collected per household. *Ae*. *aegypti* abundance outdoors was higher in lower-income neighborhoods and around older households with larger outdoor areas, greater densities of containers with standing water, less frequent yard maintenance, and greater air-conditioner use. We also found that *Ae. aegypti* abundance indoors was higher in households that had less window and door screening, less air-conditioner usage, more potted plants indoors, more rain-exposed containers around the home, and lower neighborhood human population densities. Our results indicate that, in the areas of southern California studied, there are behavioral and socio-demographic determinants of *Ae*. *aegypti* abundance, and that low-income households could be at higher risk for exposure to *Ae*. *aegypti* biting and potentially greater risk for Zika, dengue, and chikungunya virus transmission if a local outbreak were to occur.

## Author summary

Understanding the factors that influence the spread and population growth of *Aedes aegypti*, the primary mosquito vector of Zika, dengue, and chikungunya viruses, will aid vector control and public health agencies to identify the communities most at risk for *Ae*. *aegypti* biting. This study found associations between increased *Ae*. *aegypti* abundance and mappable socioeconomic variables that can serve as a basis for targeted control strategies. We describe neighborhood- and household-scale risk factors associated with increased *Ae*. *aegypti* abundance in Los Angeles, California, an area that historically has had a relatively high number of travelers return home that are infected with Zika and dengue viruses. Our results show that *Ae*. *aegypti* is more abundant in lower income communities in Los Angeles and that several household, behavioral, and socio-demographic characteristics are associated with increased abundance of *Ae*. *aegypti*. Our findings have the potential to help vector control agencies identify communities at risk of increased household *Ae*. *aegypti* abundance and develop community-specific interventions to minimize household infestations.

## Introduction

Widespread outbreaks of Zika (ZIKV), dengue (DENV) and chikungunya (CHIKV) viruses have occurred over the past decade in the Americas, including the United States. Understanding the epidemiology of these viruses for targeted surveillance and interventions to prevent future outbreaks is a top public health priority [1–9]. Since the emergence of the Zika epidemic across much of the Americas in 2015, more than 5,437 travel-associated ZIKV infections and 231 locally-acquired infections have been reported in the continental United States [10]. Local transmission of ZIKV was detected in Florida and in Texas in 2016 and 2017 with 220 and 11 locally acquired cases reported, respectively [2,3,11]. Local transmission of DENV also occurs occasionally in the continental U.S.; the most recent outbreaks were in Florida and Texas in 2013 and again in Florida in 2019, with 28, 26, and 12 locally-acquired cases reported, respectively [12]. Additionally, in 2014 along the U.S.-Mexico border, an outbreak of DENV in Sonora, Mexico resulted in 93 travel-associated cases of DENV in Arizona, with the majority of Arizona cases having recently traveled to Mexico [13]. Local transmission of CHIKV in the continental U.S. has been documented twice; in 2014, 12 locally-acquired cases of CHIKV were reported in Florida and one in Texas [14]. The explosive outbreaks of ZIKV, DENV, and CHIKV that have occurred in immunologically naïve human populations in the Americas, and the diseases’ potential for severe and life-altering clinical manifestations necessitates a robust understanding of the spatial heterogeneity of local transmission risk following virus importation in travelers in the U.S.

*Aedes aegypti* is the principal urban vector of ZIKV, DENV, and CHIKV [15–17] and has adapted to thrive in urban environments. *Ae*. *aegypti* feed frequently and primarily on humans, which makes *Ae*. *aegypti* a uniquely effective and important vector for arboviral transmission among humans [18–21]. Moreover, *Ae*. *aegypti* tend to cluster at very fine spatial scales, only dispersing short distances up to a few hundred meters, indicating that infection risk for ZIKV, DENV, and CHIKV risk similarly varies at the scale of the household [22–25]. Currently, no widely accepted vaccine for any *Ae*. *aegypti*-borne virus exists, and reducing human-*Ae*. *aegypti* contact through vector control and personal protective measures is a critically important public-health intervention to prevent ZIKV, DENV, or CHIKV transmission. There is a need to determine if those methods would similarly minimize human-*Ae*. *aegypti* contact in the arid Southwestern United States, where climatic and socio-demographic factors differ from those in the tropics where DENV is endemic.

Previous investigators have studied urban characteristics associated with DENV transmission and *Ae*. *aegypti* ecology. Poor housing conditions, high population and housing densities, and low socio-economic status (SES) have been associated with dengue in the tropics [26–29]. Similarly in the U.S., specific household characteristics and human behaviors that modify human-mosquito contact have been associated with DENV. In two studies conducted on the Texas-Mexico border, lack of air-conditioner usage was associated with higher DENV seroprevalence [8,30]. An earlier study in the Central Valley of California also showed that the attack rates of western equine encephalomyelitis virus (WEEV) and St. Louis encephalitis virus (SLEV), transmitted by *Culex tarsalis*, were negatively associated with air-conditioning and television usage, presumably because residents remained indoors behind closed windows and doors during summer evenings when *Cx*. *tarsalis* were most active [31]. The relationship between human behavior and the timing and location of mosquito biting is less clear for *Ae*. *aegypti*. Unlike *Cx*. *tarsalis*, which feed shortly after sunset and often in outdoor locations close to irrigated agriculture, *Ae*. *aegypti* feed primarily during the day and often take blood-meals inside of homes [19,25,32–35]. It is also important to consider how household characteristics influence *Ae*. *aegypti* populations. In Arizona there was no observed association between air-conditioner usage and *Ae*. *aegypti* abundance. The only factor associated with high *Ae*. *aegypti* abundance was that older houses tended to have more mosquitoes [36]. None of these studies explicitly explored how SES and neighborhood characteristics influence individual housing and human behavioral factors that modify mosquito presence or absence, such as air-conditioner usage, density of potential larval development sites, and the amount of time residents spent outside when *Ae*. *aegypti* are active. Understanding the relationships between neighborhood and household factors and *Ae*. *aegypti* immature development habitat and abundance could bolster vector control efforts by helping to identify communities that may be vulnerable to increased human-*Ae*. *aegypti* contact.

In Los Angeles County (LA), California, where an invasive population of *Ae*. *aegypti* has expanded rapidly since 2014, there is a need to target vector-control efforts to protect residents from *Ae*. *aegypti* biting. Since its first detection in the county, 82 cities and incorporated areas within LA reported *Ae*. *aegypti* infestations as of September 2019. In California, no local transmission of ZIKV, DENV, or CHIKV has been reported and no *Ae*. *aegypti* have tested positive for any of these viruses. Since 2015, however, more than 800 travel-associated cases of DENV and 700 travel-associated ZIKV infections have been reported in California, which are the highest and third highest totals among U.S. states respectively [37,38]. Since 2016, 120 travel-associated cases of CHIKV have been reported in the state [39]. Approximately 21% of travel-associated ZIKV, DENV, and CHIKV cases reported in California in 2016 and 2017 were LA residents [38–40]. LA has a highly mobile and socioeconomically diverse population with numerous immigrant communities. In 2018, 48.6% of the county’s population was Hispanic, 15.4% Asian, 9.0% African American, and 26.1% non-Hispanic, white [41]. In 2018 34.4% of LA residents were foreign-born [41]. Many of these immigrant communities maintain social connections to tropical areas that regularly experience outbreaks of *Ae*. *aegypti*-borne arboviruses [42]. LA also has a tremendous amount of inbound travel. During 2018, 7.5 million international travelers visited the county [43]. It is estimated that approximately 400 million people globally are infected with DENV every year, and that an estimated 900,000 people have been infected with ZIKV since 2015 [44,45]. Because infected travelers continue to return to parts of California with established *Ae*. *aegypti* populations, and *Ae*. *aegypti* continue to expand their habitat range, vector control agencies need tools to help them identify communities at elevated risk of *Ae*. *aegypti*-human contact that could increase risk of local outbreaks of DENV, ZIKV, or other *Ae*. *aegypti*-borne viruses.

Understanding the effect of neighborhood context on the distribution of *Ae*. *aegypti* abundance could make targeting vector control easier and more efficient because neighborhood-level data, which can be used to summarize neighborhood context (median household income and population density for example), are frequently made publicly accessible through the U.S. Census and American Community Survey. Our main objectives were to contrast the explanatory value of individual and household-level covariates as predictors of *Ae*. *aegypti* abundance with the effects of the broader neighborhood context and to identify factors associated with increased *Ae*. *aegypti* abundance in LA households. To understand these effects, we surveyed LA residents regarding their household’s demographics, behaviors, and housing conditions, and collected *Ae*. *aegypti* indoors and outdoors across six different communities. LA is an important U.S. county for understanding local *Ae*. *aegypti*-borne virus transmission and outbreak risk. It is the most populous county in the U.S., with a diverse population connected to tropical areas that have experienced ZIKV, DENV, and CHIKV outbreaks, and has a rapidly expanding population of *Ae*. *aegypti*. Here, we describe the relationship between household and neighborhood risk factors on *Ae*. *aegypti* abundance inside and outside of LA homes.

## Methods

### Ethics statement

This study was identified as exempt from federal regulations regarding human subjects research as determined by the Institutional Review Board (IRB) of the University of California, Davis (FWA: 00004557, IORG: 0000251). Written consent was obtained from all participating households.

#### Study area

To estimate the effects of income and sociodemographic factors on *Ae*. *aegypti* abundance at the household level, we interviewed residents of six socio-demographically diverse cities in LA: Boyle Heights, Commerce, East Los Angeles, Downey, La Mirada, and Whittier. Sampled households were located in census tracts with median household incomes ranging from $21,299- $136,793. These six cities represent a diverse range of incomes, and all had similar dates of initial *Ae*. *aegypti* detection; between October 2014 and August 2016 (S1 Table). LA has a Mediterranean, subtropical climate, characterized by dry summers and rainy winters. Historically, LA has received an annual average rainfall of 37.3 cm [41]. The summer dry season usually lasts from June through October. The mean daily temperature average during our sampling period was 21.0°C, with daily maximum temperatures ranging from 13.3–42.2°C, and daily minimum temperatures ranging from 7.2–29.4°C.

#### Data collection

In June and July 2017, we mailed letters in English and Spanish to 693 households. These houses were selected because the Greater Los Angeles County Vector Control District (GLACVCD) had previously identified *Ae*. *aegypti* at the properties between October 2014 –August 2016. Homes were originally inspected by technicians at GLACVCD after they had previously reported daytime biting mosquitoes to the district, or after *Ae*. *aegypti* had been identified at an adjacent neighboring home and technicians had visited them for inspections. The presence of *Ae*. *aegypti* at homes were originally confirmed by either capturing adult *Ae*. *aegypti* with handheld aspirators or by collecting immature mosquitoes and rearing and visually inspecting them at the GLACVCD lab. All interviewed homes were those where *Ae*. *aegypti* had been previously identified. Apartment buildings were excluded because it was difficult to determine which space was considered the resident’s and which was shared space with the apartment complex. A resident over 18 was required to complete the survey for participating households. In total, 7.2% (52) of households responded to the letters and were enrolled as participants. Households that did not respond to the letters were called by telephone and interviewed in either English or Spanish, as preferred. A total of 161 (23%) households were enrolled, which included those that responded to letters and those that were enrolled via phone calls.

At each household, we obtained consent to enter the resident’s private property by reading and having the participant sign a consent form in Spanish or English, verbally administered a standardized survey in Spanish or English to collect data on human behaviors and household characteristics (S1 Fig), and systematically collected adult mosquitoes. Human behaviors included activities that could expose residents to mosquito biting i.e. number of hours spent outdoors at the home, repellent use, air conditioning use, etc. Household characteristics included environmental features that could either expose residents to mosquito biting or modify larval development and adult habitat suitability, like number of potted plants, window and door screening, rain-exposed containers etc. Physical characteristics of households and surrounding areas within the property were observed and noted by the interviewer. Indoors and outdoors, we aspirated adult mosquitoes for ten minutes with a handheld electric leaf blower (Ryobi 18V ONE+, Model P2180, Anderson, South Carolina) with reversed polarity for suction and fitted with a collection cup. To ensure that we sampled when *Ae*. *aegypti* were most active, and residents were most likely at home, we surveyed between 3pm-8pm. The survey and mosquito sampling occurred once at each home during the study. All 161 enrolled households permitted outdoor aspiration, 86% (139) of households permitted indoor aspiration (Table 1). All mosquitoes were identified morphologically by visual inspection under a stereomicroscope.

**Table 1:** Summary statistics for household *Ae*. *aegypti* collections in Los Angeles, CA, August 1—October 4, 2017.

	Low income cities* (Boyle Heights, Commerce, East Los Angeles)	Medium-high income cities (Downey, La Mirada, Whittier)
**Total houses sampled**	93	68
**Houses sampled outdoors (%)**	93 (100%)	68 (100%)
**Houses sampled indoors (%)**	79 (85%)	58 (85%)
**Total *Ae*. *aegypti* collected indoors (females, males)**	20 (11, 9)	12 (10, 2)
**Total *Ae*. *aegypti* collected outdoors (females, males)**	297 (87, 204)	185 (73, 112)
**Houses with *Ae*. *aegypti* present (indoors or outdoors) (%)**	59 (63.4%)	40 (58.9%)
**Houses with *Ae*. *aegypti* indoors (%)**	11 (13.9%)	9 (15.5%)
Median household income (interquartile range (IQR))*	$42,083 ($37,242, $45,227)	$74,792 ($67,992, $87,434)
**Median census tract population density (IQR)**	18,318 (3,695.2, 21,235.3)	7,822 (6,492.6, 9,265.8)
**Median number of potted plants inside**	1–5	6–10
**Mean number of containers exposed to rain (IQR)**	20.4 (9.0, 31.0)	19.4 (8.8, 25.0)
**Mean percentage of windows and doors fully screened (IQR)**	75.0% (50.0, 100.0)	66.0% (50.0, 84.0)
**Mean hours of daily air-conditioner use (IQR)**	7.6 (2.1, 9.5)	9.3 (3.6, 12.0)
**Central air-conditioning (%)**	20.4%	72.1%
**Partial air-conditioning (window or wall units) (%)**	68.8%	17.6%
**Mean house age (years) (IQR)**	78.7 (71.0, 95.0)	61.9 (57.8, 68.0)
**Median number of containers with standing water in yard**	10 (0, 3)	10 (0, 4)
**Mean outdoor area (m**^**2**^**) (IQR)**	379 (324, 445.4)	630 (465.6, 737)
**Mean number of monthly yard** **maintenance occurrences (IQR)**	1.1 (0.0, 2.0)	2.5 (2.0, 4.0)

* We defined low income cities as those having median household incomes below the median household income for Los Angeles County, which was $64,800 in 2017 [55]. Median household income is the median of each sampled household’s census tract median household income.

We obtained census-level demographic and socioeconomic data from the 2010 United States Census and the 2011–2015 American Community Survey 5-year estimates, including population size and density, median household income, as well as race and ethnicity distributions [46]. We obtained household and property size data from the Zillow online real estate database [47], and temperature data were acquired from the Whittier Hills weather station (Station ID: GHCND:USR0000CWHH, lat/lon: 33.9839, -118.01); the closest weather station to all six cities [48].

#### Statistical analysis

We conducted a principal component analysis on all household covariates to reduce the dimensionality of the data and inform decisions regarding the combination or elimination of covariates, as the household surveys yielded 210 potential explanatory variables. Numerical covariates with highly clustered principal components or those that were collinear with other covariates and were similar in nature (e.g. window and door screening) were combined into indices as described below. In some of the surveys (<5%), participants refused to, or were unable to, answer some questions. We imputed missing covariates for this small fraction of surveys using Multivariate Imputation by Chained Equations with the *mice* package in R [49].

#### Neighborhood models

To evaluate the ability of census data alone to explain household *Ae*. *aegypti* abundance, we modeled the relationship between census tract-level covariates and household *Ae*. *aegypti* abundance. We separately analyzed the relationships between census data and *Ae*. *aegypti* abundance indoors and outdoors, using the number of *Ae*. *aegypti* collected within the 10-minute collection time indoors or outdoors as dependent variables. We compared separate models fitted for median household income and population density due to significant correlation between the two (P < 0.05), with a total of four models fitted (two each for indoor and outdoor). All models were adjusted for the mosquito collector (separately for indoor and outdoor collectors), the date of collection (to account for fluctuations in *Ae*. *aegypti* abundance), and the average daily temperature for seven days prior to the collection date, which was expected to influence *Ae*. *aegypti* development in larval habitats and survival [50,51]. To identify associations between census data and indoor and outdoor abundance, we compared model performance of nested models using the AIC and statistical significance of predictors (P < 0.05).

#### Hierarchical models

To estimate the combined effects of household characteristics and neighborhood sociodemographic characteristics, we developed multiple regression hierarchical models for indoor and outdoor *Ae*. *aegypti* household abundance that incorporated household and census tract-level covariates. These hierarchical models included fixed effects for household and census-tract-level risk factors as well as a random effect for a household’s census tract. We used a manual stepwise selection procedure with forward selection to assess model fit. As a validation method for this approach, we conducted a lasso ridge regression, a subset selection method that fits a model containing all potential coefficients and then constrains or shrinks the coefficient estimates towards zero using a penalty parameter [52]. Our inclusion criteria for covariates that remained in the final models were statistical significance (*P* < 0.05), a reduction in model AIC, and a significant likelihood ratio test against a nested model without the covariate (*P* < 0.05). After identifying eligible individual covariates, we evaluated each covariate for two-way interaction with each other covariate to assess model fit (*P* < 0.05). We tested for extra-Poisson variation by checking each model for overdispersion by comparing each model’s deviance with its residuals. Overdispersed models were modeled with a quasi-Poisson distribution and those that were not overdispersed were modeled with a Poisson distribution. All models were adjusted for the mosquito collector, date of collection, and average daily temperature during the seven days prior to the collection date. All hierarchical modeling was conducted using the *glmer* function in the *lme4* package in R version 3.6 [53].

## Results

### Survey results

**Fig 1:**
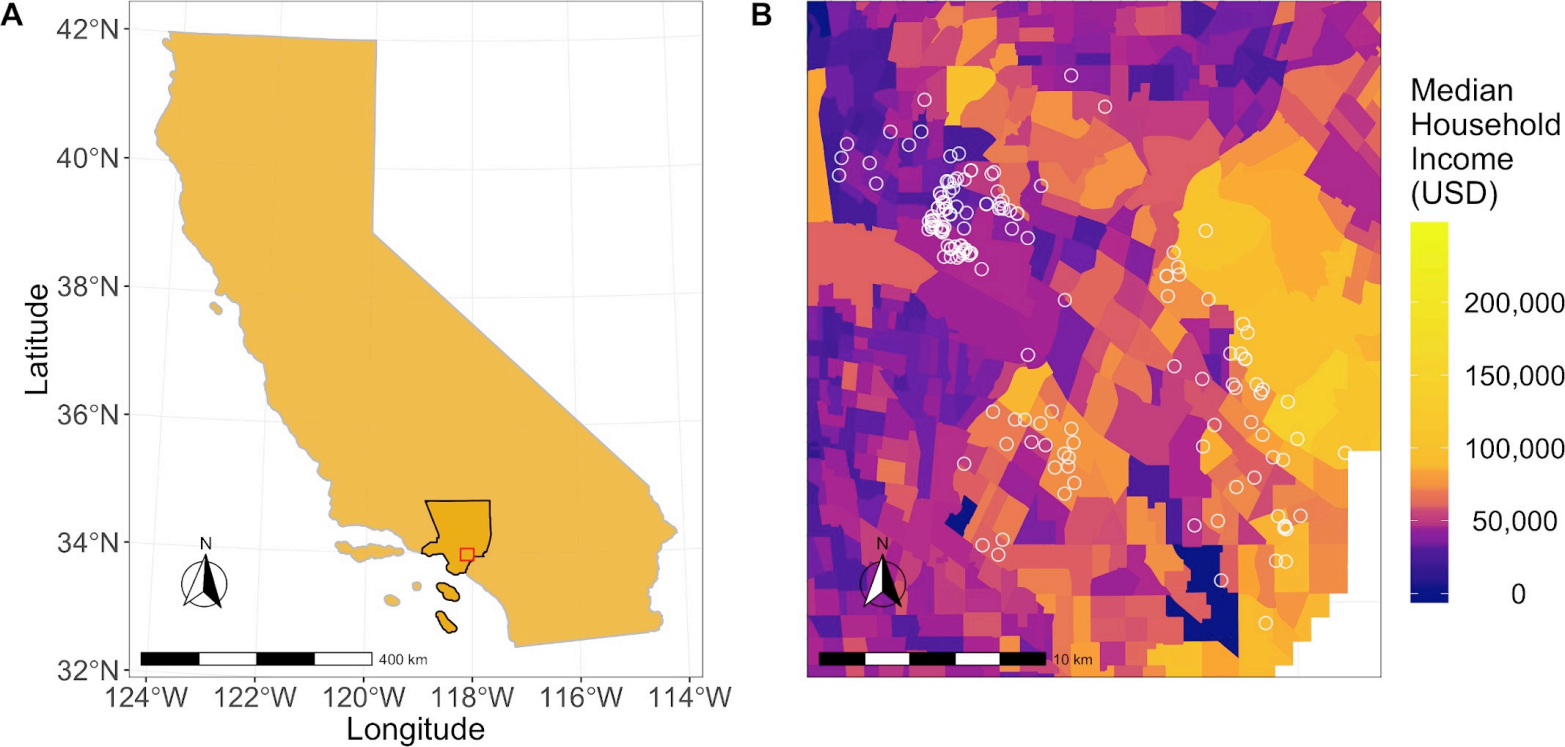
Maps showing the study area and household locations in relation to median household incomes. (A) Los Angeles County (outlined in black) and the study area (outlined in red). (B) Median household income (USD) by census tract in Los Angeles County, 2010. Geographic boundaries were obtained from the 2019 TIGER/Line shapefiles prepared by the United States Census Bureau, 2019 [56] and income values were mapped using census-tract data from the American Community Survey 5-year estimates (2012–2016) [46]. Houses surveyed for *Ae*. *aegypti* are represented by white circles.

All households were sampled between August 1 and October 4, 2017, which was reported as the period of peak *Ae*. *aegypti* abundance in LA [54]. A total of 161 households were sampled outdoors, and of those, 137 were sampled indoors. Of the sampled households, 117 (72.7%) had *Ae*. *aegypti* outdoors, and 20 of 137 (14.6%) had *Ae*. *aegypti* indoors (Table 1). The median number of *Ae*. *aegypti* we collected per household was 1, the mean was 3.1, and the range was 0–20. The sampled houses were distributed across socio-economically diverse census tracts in Los Angeles County (Figs 1 and S2 and S1 Table).

### Associations between household and sociodemographic variables

**Fig 2:**
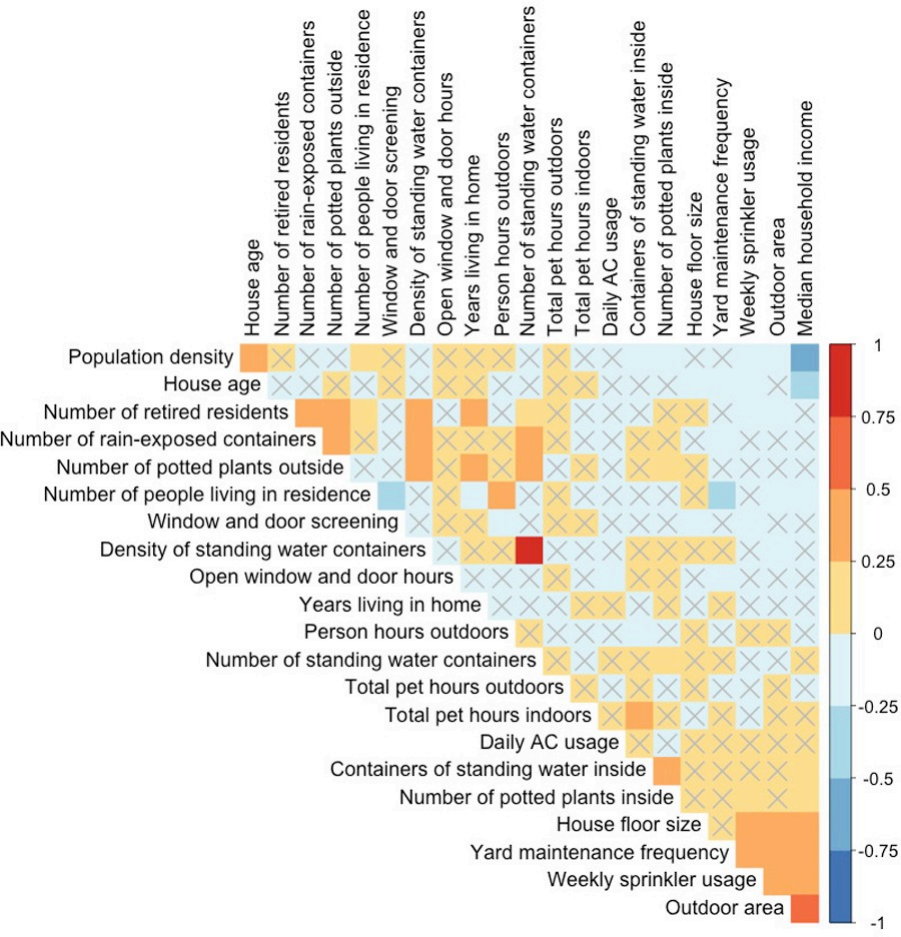
Correlations between all household and census-tract-level continuous variables among households in Los Angeles County. Colors represent the magnitude and direction of the Pearson’s correlation coefficient, respectively. Boxes without an ‘X’ indicate significantly correlated variables (*P* < 0.05).

To quantify associations between sociodemographic and household variables, we calculated Pearson’s correlation coefficients for all continuous variables (Fig 2 and S2 and S3 Tables). Higher median household income was significantly associated with greater outdoor area (*P* < 0.001), larger home size (*P* = 0.001), more frequent sprinkler usage (*P* < 0.001), more frequent lawn maintenance (*P* < 0.001), more indoor potted plants (*P* = 0.001), and more containers with standing water indoors (*P* = 0.04). Conversely, lower median household income was significantly associated with more people per household (*P* = 0.01), older homes (*P* < 0.001), and a higher population density (*P* < 0.001).

### Neighborhood-level predictors of *Ae*. *aegypti* abundance

**Fig 3:**
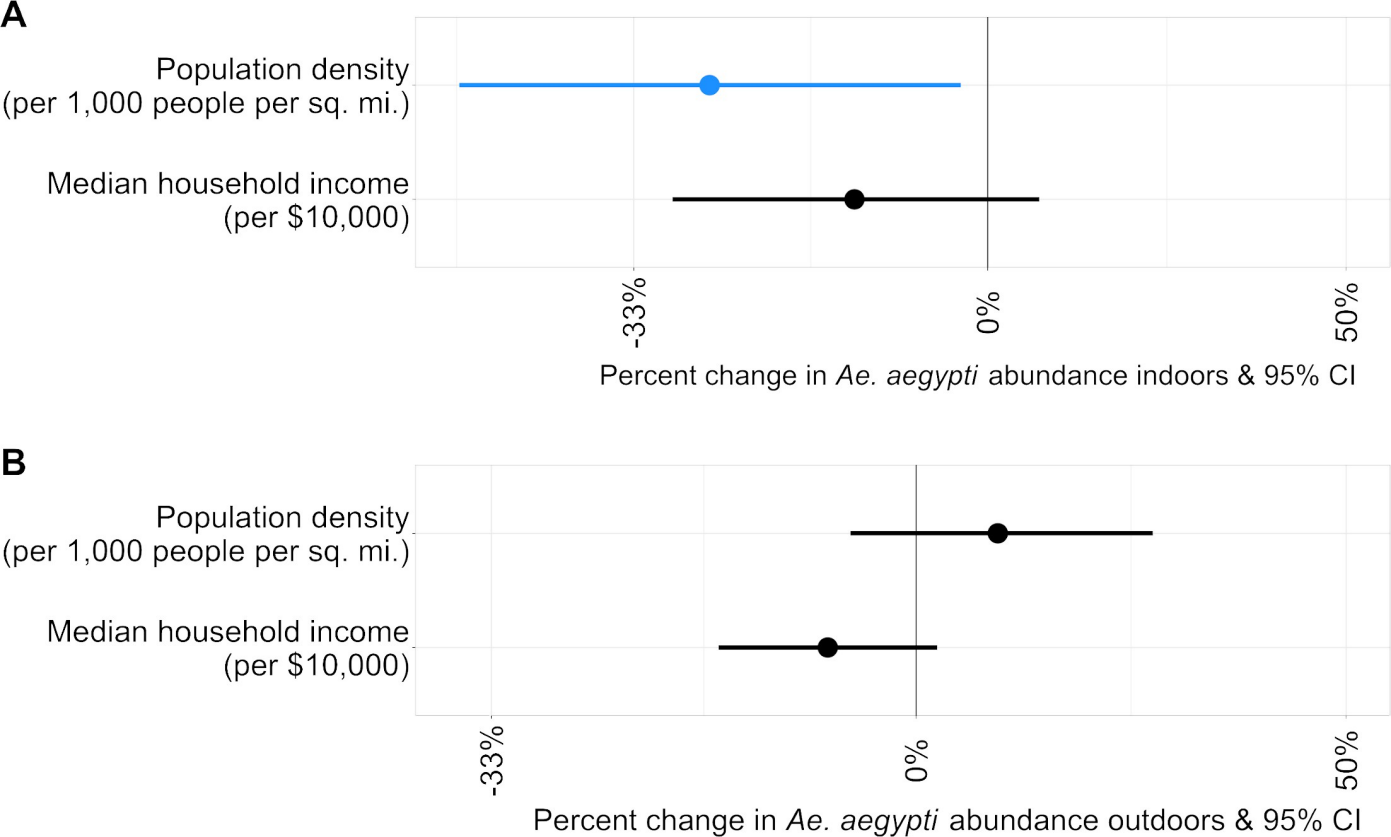
Rate ratios for census-tract-level predictors of *Ae*. *aegypti* detections. (A) Rate ratios for *Ae*. *aegypti* collected indoors. (B) Rate ratios for *Ae*. *aegypti* collected outdoors. Models for outdoor *Ae*. *aegypti* counts were quasi-Poisson regression models, models for *Ae*. *aegypti* counts indoors were Poisson regression models. For clarity, rate ratios are expressed as a percentage change for each covariate. All models were adjusted for the mosquito collector, average daily temperature of the seven days prior to collection, and the collection date. Blue indicates a rate ratio < 1, and black indicates a non-significant predictor (*P* > 0.05). X-axis is on a logarithmic scale.

To estimate associations between socioeconomic risk factors and household *Ae*. *aegypti* abundance, we included census-tract sociodemographic data as covariates in multiple regression models (Fig 3 and S4 and S5 Tables). Median household income alone was not associated significantly with *Ae*. *aegypti* abundance outdoors or indoors (*P* = 0.134, 0.159 respectively). Census tract population density also was not significantly associated with *Ae*. *aegypti* abundance outdoors (*P* = 0.281). However, lower population density was associated with greater *Ae*. *aegypti* abundance indoors (rate ratio = 0.73; *P* = 0.032). An increase of 5,000 people per square mile corresponded to a 27% lower abundance of indoor *Ae*. *aegypti*.

### Combined effects of neighborhood- and household-level predictors on *Ae*. *aegypti* abundance

**Fig 4:**
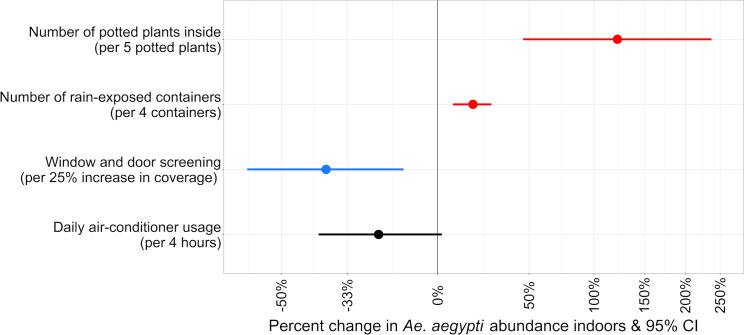
Rate ratios for census-tract and household-level predictors included in final multiple Poisson regression model for *Ae*. *aegypti* detections indoors. For clarity, rate ratios are expressed as a percentage change for each covariate. The model was adjusted for mosquito collector, average daily temperature of the seven days prior to collection, and collection date. Red indicates a risk factor (P < 0.05), blue indicates a protective behavior (P < 0.05), and black indicates a non-significant predictor (P > 0.05) that significantly improved model fit (P < 0.05). X-axis is on the logarithmic scale.

**Fig 5:**
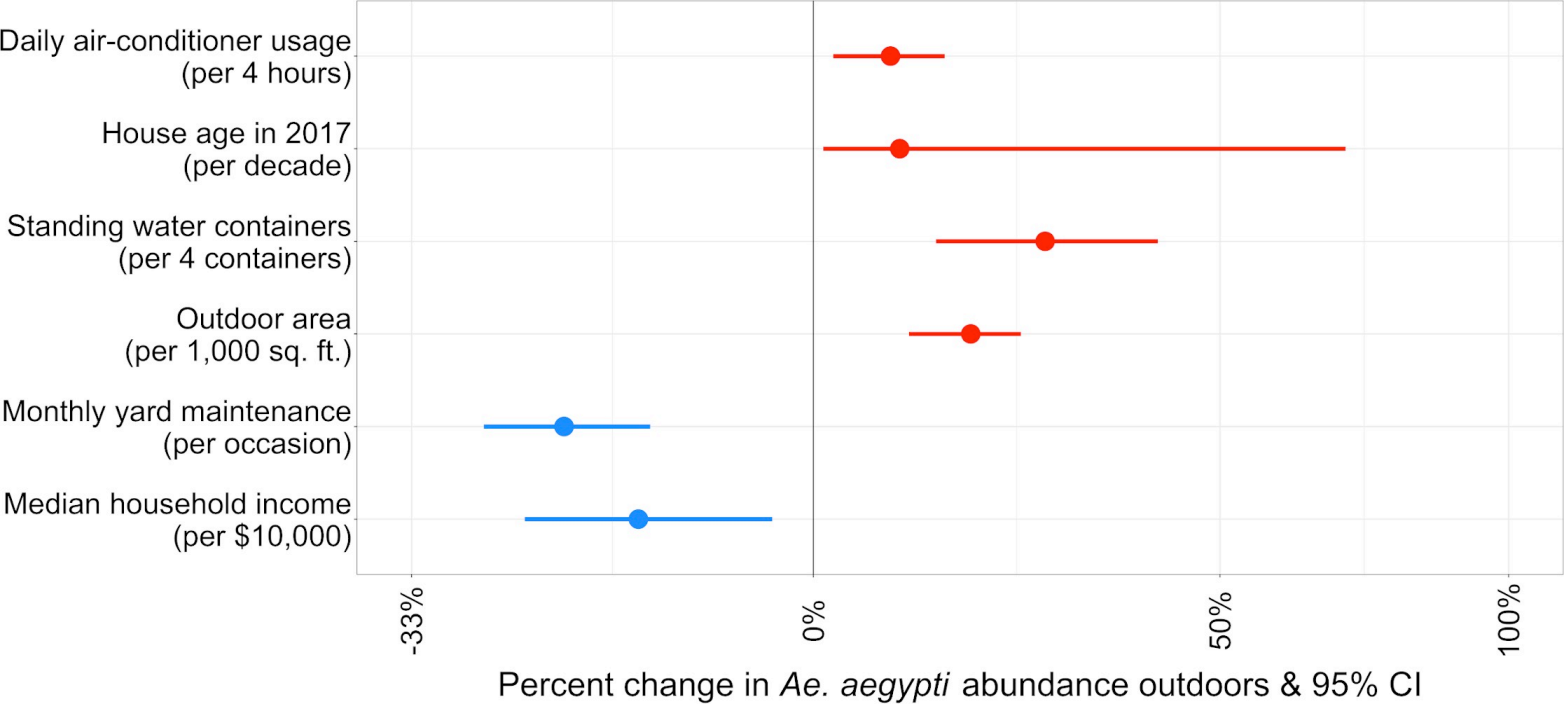
**Rate ratios for census-tract and household-level predictors included in final multiple hierarchical Poisson regression model for *Ae*. *aegypti* detections outdoors.** For clarity, rate ratios are expressed as a percentage change for each covariate. The model was adjusted for mosquito collector, average daily temperature of the seven days prior to collection, and collection date. Red indicates a risk factor (P < 0.05) and blue indicates a protective behavior (P < 0.05). X-axis is on the logarithmic scale.

Hierarchical models were fitted to estimate *Ae*. *aegypti* counts as a function of the combined effects of household characteristics and neighborhood socio-demographics. It was not possible to fit a hierarchical model for indoor abundance due to the small number of *Ae*. *aegypti* caught indoors. We, therefore, present the non-hierarchical model for indoor *Ae*. *aegypti* abundance, which examines only household predictors (Fig 4 and S6 Table), along with the results from the hierarchical model for outdoor *Ae*. *aegypti* abundance, which examines household and neighborhood-level predictors (Fig 5 and S7 Table). The lasso ridge regression yielded a set of statistically significant coefficients (*P <* 0.05) similar to the final models fitted with the stepwise approach (S3 Fig). Therefore, we present results from the stepwise approach only. We did not consider interactions between variables in the model because of sample size and power limitations and to maximize interpretability of covariate associations for public health and vector control operations. *Aedes aegypti* abundance indoors was significantly higher among households with more potted plants indoors or more rain-exposed containers outside the home (*P* < 0.05). Rain-exposed containers were defined as any type of object that was not covered by a structure (e.g. patio, garage, etc.) that was either holding liquid or had the potential to hold liquid if it rained or received water from a lawn sprinkler, including objects like buckets, toys, dishes, bottle caps, tarps, rubbish etc. Indoor abundance was also significantly lower among households with higher proportions of window and door screening (*P* < 0.05). An increase of five potted plants indoors corresponded to a 122% increase in the number of indoor *Ae*. *aegypti* (*P* < 0.001), while an increase of four rain-exposed containers on the property corresponded to a 17% increase in the number of *Ae*. *aegypti* indoors (*P* < 0.001). A 25% increase in screening of windows and doors was associated with a 39% reduction in the number of *Ae*. *aegypti* collected indoors (*P* = 0.006). Air-conditioning usage was just above our threshold for inclusion (*P* = 0.064), however, it significantly improved model fit (P < 0.05) and was kept in the final model. For a four-hour increase in the number of hours of air-conditioning use each day, indoor *Ae*. *aegypti* abundance declined by 27%.

Outdoor *Ae*. *aegypti* abundance was significantly higher among households that were older, had larger outdoor areas, higher densities of containers with standing water, and those that were located in census tracts with lower median household income. In LA, for a 10-year increase in home age, *Ae*. *aegypti* abundance increased by 9% (*P* = 0.029). For every 100 m^2^ increase in outdoor space, outdoor *Ae*. *aegypti* abundance increased by 14% (*P* < 0.001). An increase of one container per 100 m^2^ corresponded to an increase in *Ae*. *aegypti* abundance of 25% (*P* < 0.001) and every increase in annual income of $10,000 corresponded with a reduction in outdoor *Ae*. *aegypti* abundance of 16% (*P* = 0.009). *Aedes aegypti* abundance decreased by 22% with each monthly instance of yard maintenance (*P* < 0.001) and increased by 8% for every four hour increase in the number of hours air-conditioning was used each day in a given household (*P* = 0.006). All variables included in the final hierarchical model for *Ae*. *aegypti* outdoors were statistically significant (*P* < 0.05).

## Discussion

Managing mosquito infestations and individual household surveillance are important components of preventing and minimizing outbreaks of ZIKV, DENV, and CHIKV. As invasive *Ae*. *aegypti* continue to spread North in California and elsewhere in the U.S., and travelers continue to move between regions of the world where ZIKV, DENV, or CHIKV are transmitted, identifying neighborhoods with high human-*Ae*. *aegypti* contact is important information for preventing and mitigating local outbreaks. In California, vector biologists and technicians routinely respond to household service requests after reports of day-biting mosquitoes and conduct intensive surveillance in neighborhoods where imported ZIKV or DENV cases reside. Novel methods to identify neighborhoods and households at risk for human-*Ae*. *aegypti* contact before infestations become established and cases are reported would improve current California vector control efforts, especially because in California, identification of new *Aedes spp*. infestations often occur well after the infestation has begun [57,58]. In this study, we found that abundance was higher in lower-income neighborhoods and older households with more larval development and resting mosquito habitat.

*Aedes aegypti* abundance indoors and outdoors varied in proportion to human density and land area, respectively, which indicated that the built environment and neighborhood layout influenced *Ae*. *aegypti* habitat suitability. Increased population density was associated with reduced *Ae*. *aegypti* abundance indoors. The negative correlations between population density and potted plants and containers of standing water indoors could explain this finding. Areas with high human population density may have less suitable habitat for *Ae*. *aegypti* indoors than areas with low population density, assuming regular service and waste removal reduces availability of suitable habitats. Although human population density was significantly associated with indoor *Ae*. *aegypti* abundance, it was not associated with *Ae*. *aegypti* abundance outdoors. Researchers in Tucson, AZ similarly found that human population density did not predict *Ae*. *aegypti* abundance outdoors [36]. The communities we surveyed are all considered to be urban, and it could be that the population densities captured in our study did not span a wide enough range to capture the broader effects of population density on *Ae*. *aegypti* abundance. *Ae*. *aegypti* typically fly no more that 100–300 meters, and because of this there could be population or housing density thresholds which could potentially limit population spread or growth due to host or habitat availability [22–25]. To understand these effects, future studies should aim to sample a great range of population and housing densities. Taken together, our findings indicate that human population density could be used by vector control agencies to identify neighborhoods that are at increased risk of indoor *Ae*. *aegypti* exposure. This could be used in conjunction with the household characteristics we described to estimate risk for outdoor *Ae*. *aegypti* infestation and mosquito population spread. Increased outdoor property area was the household characteristic most strongly associated with increases in outdoor *Ae*. *aegypti* abundance. This is the first study to describe a positive association between outdoor area and increased *Ae*. *aegypti* abundance. We found that smaller lot sizes were more commonly associated with older houses and greater house age was also associated with increased *Ae*. *aegypti* outdoors, possibly due to a cumulative increase in the number of larval development sites on those properties over time. Paradoxically, larger lot size might also influence *Ae*. *aegypti* population dynamics by providing more shaded resting habitat for adult mosquitoes, increased vegetation for sugar-feeding, or unrecognized larval development sites due to the increased size of some yards. In Arizona, increased vegetation was associated with increases in *Ae*. *aegypti* abundance, and in Ecuador, shaded patios significantly increased DENV infection risk [59,60]. Although outdoor area was not significantly correlated with the number of outdoor potted plants in our study, it was positively associated with more frequent yard maintenance and sprinkler usage, which could contribute to vegetation abundance, water accumulation in unrecognized containers, or increased humidity and subsequently lead to increases in larval development sites or adult survival. Future studies should examine the relationship between a property’s outdoor area, vegetation abundance (for example, via remote sensing) and type, and *Ae*. *aegypti* resting habitat and sugar-feeding to determine the extent to which vegetation is associated with property size and adult *Ae*. *aegypti* abundance and survival.

Even though LA has little to no rainfall during the summer when *Ae*. *aegypti* abundance is highest, several significant associations related to *Ae*. *aegypti* abundance indoors and outdoors were related to larval habitat availability, indicating that water management practices around the home influence *Ae*. *aegypti* abundance in LA and possibly elsewhere in the arid southwestern U.S. Among the sampled LA households, the majority containers of standing water were often objects that had accumulated water due to watering practices (e.g. potted plant dishes, watering cans or toys and rubbish that had been left in yards and irrigated by sprinklers), and the minority were water storage vessels (e.g. buckets or rain water basins). All observed rainwater storage vessels were covered with a lid. The number of containers holding standing water outdoors was not associated with *Ae*. *aegypti* abundance. Houses with greater densities of outdoor standing water containers and older homes were associated with greater *Ae*. *aegypti* abundance outdoors. This indicates that households with smaller lot sizes and more standing water may have greater *Ae*. *aegypti* abundance. In Arizona, older homes had greater *Ae*. *aegypti* abundance, possibly by providing more suitable habitat for *Ae*. *aegypti* via accumulated deterioration and settling that leads to an accumulation of larval development sites [36]. Older homes in our study tended to have a lower household income and reported less frequent yard maintenance than newer homes. The financial barriers to yard maintenance faced by some lower income families is one possible explanation for how social determinants of health could increase risk of *Ae*. *aegypti* exposure in the U.S. Available data on housing construction dates are publicly available on platforms like Zillow, and home age could prove useful for targeting vector control agency interventions.

The two strongest associations with increased *Ae*. *aegypti* abundance indoors were high quantities of rain-exposed containers outdoors and high quantities of potted plants indoors. The number of rain-exposed containers was significantly positively correlated with high quantities of containers with standing water, higher standing water densities, and the number of potted plants outside, all of which provide larval development, resting habitat, and sugar-feeding sources for adult mosquitoes. Trash in and around homes has been associated with *Ae*. *aegypti* abundance internationally [61–64]. In California, vector control technicians anecdotally reported having observed more mosquitoes at properties with more waste and other discarded objects in yards, which may indicate that yards with these characteristics provide more suitable larval development sites and resting habitat for adults in LA. Accumulation of trash at the home could be the result of financial barriers to obtaining trash hauling or maintenance services, time constraints due to occupational or familial responsibilities, or a failure of private or municipal services to adequately dispose of them. Although the correlation between rain-exposed containers and window and door screening was not statistically significant (*P* > 0.05), the direction of this relationship (Pearson’s correlation coefficient = -0.07) indicates that houses with more rain-exposed containers may have fewer screens, which could contribute to mosquitoes more easily entering these homes. Unfortunately, we did not quantify household trash or accumulated yard waste in this study. Investigating the relationship between *Ae*. *aegypti* abundance, rain-exposed containers, and trash in the United States is an important next step for researchers attempting to evaluate the impacts of social determinants of health on *Ae*. *aegypti* abundance.

Our results show that efforts to physically prevent mosquitoes from entering the home were associated with a reduction in *Ae*. *aegypti* abundance indoors, indicating that households facing barriers to home improvements may have an increased risk of indoor *Ae*. *aegypti* infestations. We found that more frequent use of air-conditioning was associated with fewer *Ae*. *aegypti* indoors. Conversely, and unexpectedly, these results suggest that households with increased air-conditioner usage also had an increased abundance of *Ae*. *aegypti* outdoors. In Tucson, AZ, Hayden *et al*. found that the presence of air-conditioning was associated with decreases in *Ae*. *aegypti* abundance outdoors, however in Nogales, AZ, they found that the presence of swamp coolers (a device that cools air via evaporative cooling) was a significant risk factor for increased outdoor abundance [59]. We explored differences between the presence and absence of central and non-central air-conditioning (i.e. window or wall units) but found no statistically significant association (*P* > 0.05). Other studies conducted along the Texas-Mexico border have found that air-conditioner presence was associated with decreases in DENV infection, likely by preventing *Ae*. *aegypti* from entering the home [8,30,64]. One possible explanation is that increased air-conditioner use may be related to household characteristics and human behaviors (e.g. vegetation, lack of time spent outdoors and therefore less maintenance or noticing of standing water) that could increase the number of breeding *Ae*. *aegypti* or resting habitat availability for the mosquitoes. Although window and door screens seem to physically prevent *Ae*. *aegypti* from entering homes, air-conditioner usage could also contribute to indoor temperatures that are cooler than the optimum for *Ae*. *aegypti*. Air-conditioner use may not lead to reductions in *Ae*. *aegypti* abundance at the household level, but probably also protects humans from mosquito bites indoors. Financial barriers preventing households from using air-conditioners and maintaining window screens could lead to increased *Ae*. *aegypti* exposure inside LA homes. Future research should investigate how different types of air-conditioning, i.e. central, widow units, swamp coolers, etc., influence *Ae*. *aegypti* ecology.

We found that households in wealthier neighborhoods had fewer *Ae*. *aegypti* outdoors than households in areas with lower incomes, which is consistent previous findings from other countries that *Ae*. *aegypti* disproportionately thrive in low-income areas [65–69]. Although median household income alone was not significantly associated with outdoor abundance (*P* > 0.05), we found an association between wealthier areas and fewer *Ae*. *aegypti* outdoors after accounting for individual household variables (*P* = 0.007). Poverty is closely related to household characteristics and human behavioral traits that influence *Ae*. *aegypti* biology and access to blood-meals. Studies conducted elsewhere in the world have reported robust evidence that poverty is associated with increased *Ae*. *aegypti* abundance [65–69], and our study is one of the first to suggest this in the continental U.S. [36] and the first in the newly invaded areas of the arid Pacific Southwest. Poverty is one of the most well-established and robust risk factors for many infectious and non-infectious diseases [70–75], and even within regions of high absolute poverty, like urban slums in Brazil, dengue risk has been shown to increase with increasing poverty; disease burden is highest in the poorest groups and the most vulnerable populations [29]. Additionally, numerous other studies across the world have shown that low socioeconomic status is closely related to increased DENV transmission and human population seroprevalence [26–28,30,36,64,76,77]. While the presence of *Ae*. *aegypti* does not explicitly lead to ZIKV, DENV or CHIKV infection, the cumulative life experiences and subsequent epigenetic profiles of some residents of low-income communities we studied may put these populations at greater risk for exposure to *Ae*. *aegypti* and the development of symptomatic disease, especially if their low-income status is accompanied by limited access to healthcare and health education and information. We studied several low income communities of East Los Angeles, Commerce, and Boyle Heights, all of which are predominantly Latino communities with more population exchange and travel to regions of the world where DENV is endemic and where ZIKV was heavily transmitted during the last epidemic wave [78,79]. These connections could put these communities at greater risk of having an introduction event of ZIKV or DENV. Coupled with greater *Ae*. *aegypti* abundance, these communities are likely at higher risk for local transmission than higher income communities in LA. Lower income communities also tend to be more disenfranchised with more barriers to accessing healthcare and services. Our results, paired with the findings of others, suggest the need for enhanced vector control and outreach in communities with relatively lower socioeconomic status to limit the risk of invasive *Ae*. *aegypti* spread and local transmission of ZIKV, DENV, CHIKV, and other *Aedes sp*.*-*transmitted viruses.

There were several limitations to our study. One limitation of this study was that we only enrolled participants from households that had previous contact with the GLACVCD. This may have biased estimated effects by narrowing the range of enrolled households to those that had elevated mosquito abundance or tended to be more vigilant about noticing or controlling mosquitoes. Another limitation to our study was that there were too few mosquitoes captured indoors to build a robust hierarchical model to evaluate the relationship between *Ae*. *aegypti* abundance indoors, and any of our risk factors of interest. It is also possible that because we only included ecological census data in the census models, the observed relationship between indoor abundance and population density was due to unmeasured covariates. Future studies should enroll larger samples to produce more robust estimates. Although we included daily average temperature for the previous seven days as a potential confounder in all of our models, these temperature data came from a single weather station nearby and not individual households. We did not incorporate other climatic variables that have the potential to influence *Ae*. *aegypti* population dynamics, like humidity and vapor pressure, as these data are hard to quantify and interpret at the household scale. Collecting and incorporating these climate variables at the household scale in future studies may yield more precise relationships between household characteristics and *Ae*. *aegypti* abundance. Additionally, we did not categorize the various types of containers that held standing water and counted all types of containers together. Future studies should aim to characterize the distribution of types of standing water containers, which could have important implications for *Ae*. *aegypti* productivity and immature habitat availability. As a cross-sectional study, the associations described herein lack temporality, and future research should investigate the longitudinal associations in these communities prior to large-scale implementation of mosquito control interventions.

Our results indicate that there were poverty-associated social and environmental determinants of *Ae*. *aegypti* abundance and that people living in low-income communities in Los Angeles, California were at higher risk for *Ae*. *aegypti* exposure. They also indicate that specific control measures targeted at the household level could reduce the abundance of *Ae*. *aegypti* in LA. We recommend that future studies investigate intervention methods for reducing *Ae*. *aegypti* populations, specifically evaluating the impact of increasing yard maintenance, decreasing standing water density, reducing the number of potted plants inside and the number of rain-exposed containers on properties, increasing window and door screening, and increasing air-conditioner usage particularly in low-income communities. The associations between air-conditioner use, income, and *Ae*. *aegypti* abundance differed between studies conducted in LA, Arizona, and Texas, indicating that a universal approach to predicting or controlling *Ae*. *aegypti* abundance in the U.S. may not be a realistic goal. We, therefore, recommend that vector control districts and policy makers in the U.S. continue to invest in developing connections with communities and practicing local engagement to develop community-specific strategies optimized for specific microclimatic, cultural, and population patterns as recommended by the World Health Organization [80]. As climate change increases the range of suitable habitat for *Ae*. *aegypti* it is likely that the range of DENV transmission will follow suit, and possible that ZIKV and DENV outbreaks will also occur in the future [81–90]. With a changing climate, these community specific engagement efforts and advanced preparation to prevent infestations take on greater urgency. If these trends continue to expand the range of *Ae*. *aegypti* in the U.S., factors like frequency of air-conditioning and yard maintenance may become important drivers of heterogeneity in *Ae*. *aegypti* abundance and exposure. As travelers infected with ZIKV or DENV continue to return to and visit California, and *Ae*. *aegypti* and the viruses they transmit continue to spread, models like those presented here will be important for the identification of communities at risk for local transmission and prioritizing vector control mitigation and community outreach.

## Supporting information

S1 Fig The survey administered to all participants. During the household visit, one researcher would ask the participant questions from the survey in English or in Spanish and record the participant’s responses on this document.
(PDF)

S2 Fig Comparison of median household income distribution, in US dollars, by census tract in Los Angeles County (yellow) and surveyed households (purple), 2010. Values are based on census-tract data from the American Community Survey 5-year estimates (2011–2016)[44].
(DOCX)

S3 Fig Standardized lasso coefficients for *Ae*. *aegypti* abundance measured outdoors. (DOCX)

S1 Table Income and population summary statistics for cities surveyed in Los Angeles County, California. Census-tract data were collected from the American Community Survey 5-year estimates (2011–2016). (DOCX)

S2 Table Household and census-tract level predictors of *Ae*. *aegypti* abundance indoors and outdoors used in Poisson and quasi-Poisson models. Household data were collected during the household surveys (2017) and census-tract data were collected from the American Community Survey 5-year estimates (2011–2016).
(DOCX)

S3 Table Pearson’s correlation coefficients for all household and census tract level continuous variables among households in Los Angeles County surveyed for *Ae*. *aegypti* in 2017. The order of pairs corresponds to the order of pairs presented in Fig 2.
(DOCX)

S4 Table Rate ratios for census-tract-level predictors of *Ae*. *aegypti* detections indoors. Models for *Ae*. *aegypti* counts indoors are Poisson regression models. Rate ratios and 95% confidence intervals are shown for all census-tract-level predictors included in the census-tract models. These models were adjusted for the mosquito collector, average daily temperature of the seven days prior to collection, and the collection date.
(DOCX)

S5 Table Rate ratios for census-tract-level predictors of *Ae*. *aegypti* detections outdoors. Models for outdoor *Ae*. *aegypti* counts were quasi-Poisson regression models. Rate ratios and 95% confidence intervals are shown for all census-tract-level predictors included in the census-tract models. These models were adjusted for the mosquito collector, average daily temperature of the seven days prior to collection, and the collection date.
(DOCX)

S6 Table Rate ratios from the Poisson regression model for *Ae*. *aegypti* counts indoors. Rate ratios and 95% confidence intervals are shown for all household predictors included in the model. This model was adjusted for the mosquito collector, average daily temperature of the seven days prior to collection, and the collection date.
(DOCX)

S7 Table Rate ratios from the hierarchical Poisson regression model for *Ae*. *aegypti* counts outdoors with a random variable for census tract. Rate ratios and 95% confidence intervals are shown for all household and census-level predictors included in the model. This model was adjusted for the mosquito collector, average daily temperature of the seven days prior to collection, and the collection date.
(DOCX)

S1 Dataset Data from participating households in Los Angeles County, CA. Data were obtained from surveys conducted during August-October 2017 and from the American Community Survey 5-year estimates, 2012–2016.
(CSV)

S1 File Data dictionary for data presented in Dataset S1. (CSV)
