# Supplementary material for: Quantifying sociodemographic heterogeneities in the distribution of Aedes aegypti among California households
Source: PLoS Negl Trop Dis. 2020 Jul 21;14(7):e0008408. doi: 10.1371/journal.pntd.0008408 (PMC7394445; doi:10.1371/journal.pntd.0008408)
Supplement: S1 Fig — During the household visit, one researcher would ask the participant questions from the survey in English or in Spanish and record the participant’s responses on this document. (PDF) [file pntd.0008408.s001.pdf]

Los Angeles County Mosquito Survey

Date of Visit (mm/dd/yy): ____________
Collection ID:______________ **Occupant:** _______________________
Address: ____
Telephone:______________________
Collector:______________________ Surveyor:______________________
Collection time (e.g. 3:30 pm):______________
Human behavior:
Does anyone at the home get bit by mosquitoes? □Yes □No □Don't know
How long have residents lived in this home? ______ How many people live at this residence? ______
Number of people typically at home:
Weekdays (Mon-Fri or other work days): Weekends (Sat-Sun or other non-work days):
Day (8am-5pm):______ Night (5pm-8am):_______ Day (8am-5pm):______ Night (5pm-8am):_______

How many hours does each person spent **outdoors** at home? (Person-hours, cumulative for all household members):
(For each person, add up the no. of hours each person spends outdoors at home to calculate person-hours)

Weekday Daytime (8am-5pm):	Weekday Nighttime (5pm-8am):	Weekend daytime (8am-5pm):	Weekend Nighttime (5pm-8am):

Weekdays (Mon-Fri or other work days): Weekends (Sat-Sun or other non-work days):
Total Day person-hours:_______ Total Day person-hours:_______
Total Night person-hours:_______ Total Night person-hours:_______
How many people in the household are home-bound or retired? (Spend most time at home) _______
Household behaviors:
No. of bedrooms: ____ No. of all rooms (including bedrooms):_____ No. of windows (that open): _____
No. of windows with intact screens:____
No. of exterior doors: _____ Do these doors have intact screens on them? □Yes □No
No. of windows open during: Day: _____ Evening: _____ Non-work day: Day: _____ Evening: _____
Time frame windows are open (e.g. 8-10am): Day: _________ Evening:_________ Non-work day: Day:_________ Evening: _________
No. of doors open during (with or without screens): Day:_____ Evening:_____ Non-work day: Day: _____ Evening: _____
Time frame doors are open (e.g. 8-10am): Day:_________ Evening:_________ Non-work day: Day: _________ Evening: _________
Air-conditioning: □ None □ Central □ Window unit(s) □ Other: ____________________
If air-conditioning present, total number of hours air-conditioning is on:
Weekdays (Mon-Fri or other work days): Weekends (Sat-Sun or other non-work days):
Day (8am-5pm):______ Night (5pm-8am):_______ Day (8am-5pm):______ Night (5pm-8am):_______
Do you store water? (Not water bottles) □Yes □No If Yes: □Indoors □Outdoors Is it: □Covered □Uncovered
Do you have gutters? □Yes □No If Yes, do you clean your gutters (or have someone else do it)? □Yes □No
How many times per year? ____

Does someone come to your house to do professional yard maintenance? □Yes □No If Yes, how many times per month?______

Is there a sprinkler system present? □Yes □No If Yes, how many times per week is it on?______

Do you perform any mosquito control in your house at your own expense? □Yes □No

If Yes, date of last application:__________

Type of control:____

Do household members ever wear repellent? □ All of them do □ Some of them do □ None of them do

Animals on the property: □No animals present

	How many?	Hours spent indoors	Hours spent outdoors
Dogs			
Cats			
Chickens			
Other: __________________			
Other: __________________			

Outside house characteristics:

No. of potted plants outside house (in yard): □ 0 □ 1-5 □ 6-10 □ 11-15 □16-20 □ 20+

No. of containers in yard that do or have the potential to collect water (i.e. fountains, toys, trash, laundry buckets etc.): □ 0 □1-5 □6-10 □ 11-15 □16-20 □ 20+

How many of these containers are exposed to rain (uncovered and not under shelters)? ______ (Estimate)

How many containers have standing water in them?__________

Are there bushes/shrubs in the yard? □Yes □No

Are there trees in the yard? □Yes □No

Is there grass in the yard? □Yes □No

Is there a pool at the house? □Yes □No If Yes, is it full? □Yes □No

If full, is it clean? □Clean □Mostly clean □Not clean

Lawn drains present? □Yes □No If Yes: □Covered □Uncovered

House drainpipe present? □Yes □No If Yes, is it: □Covered □Uncovered

Inside house characteristics:

No. of potted plants inside house: □ 0 □1-5 □ 6-10 □ 11-15 □16-20 □ 20+

No. of containers inside house that store water (i.e. dog bowls, fountains, toys etc.):

□ 0 □1-5 □6-10 □11-15 □16-20 □ 20+

Mosquito Collection (adults):

Estimated area of the house (ft^2): __________ Estimated area of the yard (front and back, ft^2):__________

Collection duration (in minutes) for adults: Inside:__________ Outside:__________

Did you identify mosquito larvae or pupae in water sources around the house or yard? □ Yes □ No

How many containers had larvae or pupae in them? Inside:__________ Outside: __________

No. of adult *Ae. aegypti* found in lawn drain: __________

Notes/Issues that arose during survey:

For after the survey:

Were there any *Ae. aegypti* collected at the house? If yes, how many were collected:

Inside? Males ______ Females_______ Outside? Males ______ Females_______

How many of these were blood-engorged? Inside______ Outside______
