# Supplementary material for: Quantifying sociodemographic heterogeneities in the distribution of Aedes aegypti among California households
Source: PLoS Negl Trop Dis. 2020 Jul 21;14(7):e0008408. doi: 10.1371/journal.pntd.0008408 (PMC7394445; doi:10.1371/journal.pntd.0008408)
Supplement: S2 Fig — Values are based on census-tract data from the American Community Survey 5-year estimates (2011–2016)[44]. (DOCX) [file pntd.0008408.s002.docx]

**
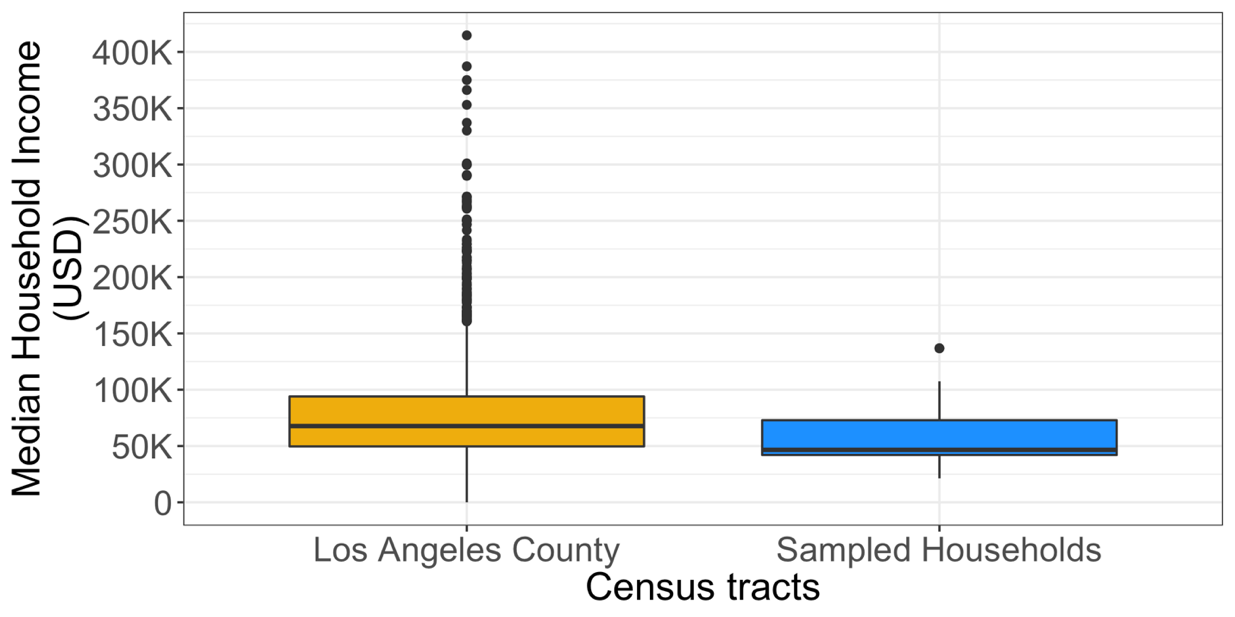
**

**Fig S2:** Comparison of median household income distribution, in US dollars, by census tract in Los Angeles County (yellow) and surveyed households (purple), 2010. Values are based on census-tract data from the American Community Survey 5-year estimates (2011-2016).
