# Supplementary material for: Quantifying sociodemographic heterogeneities in the distribution of Aedes aegypti among California households
Source: PLoS Negl Trop Dis. 2020 Jul 21;14(7):e0008408. doi: 10.1371/journal.pntd.0008408 (PMC7394445; doi:10.1371/journal.pntd.0008408)
Supplement: S3 Fig — (DOCX) [file pntd.0008408.s003.docx]

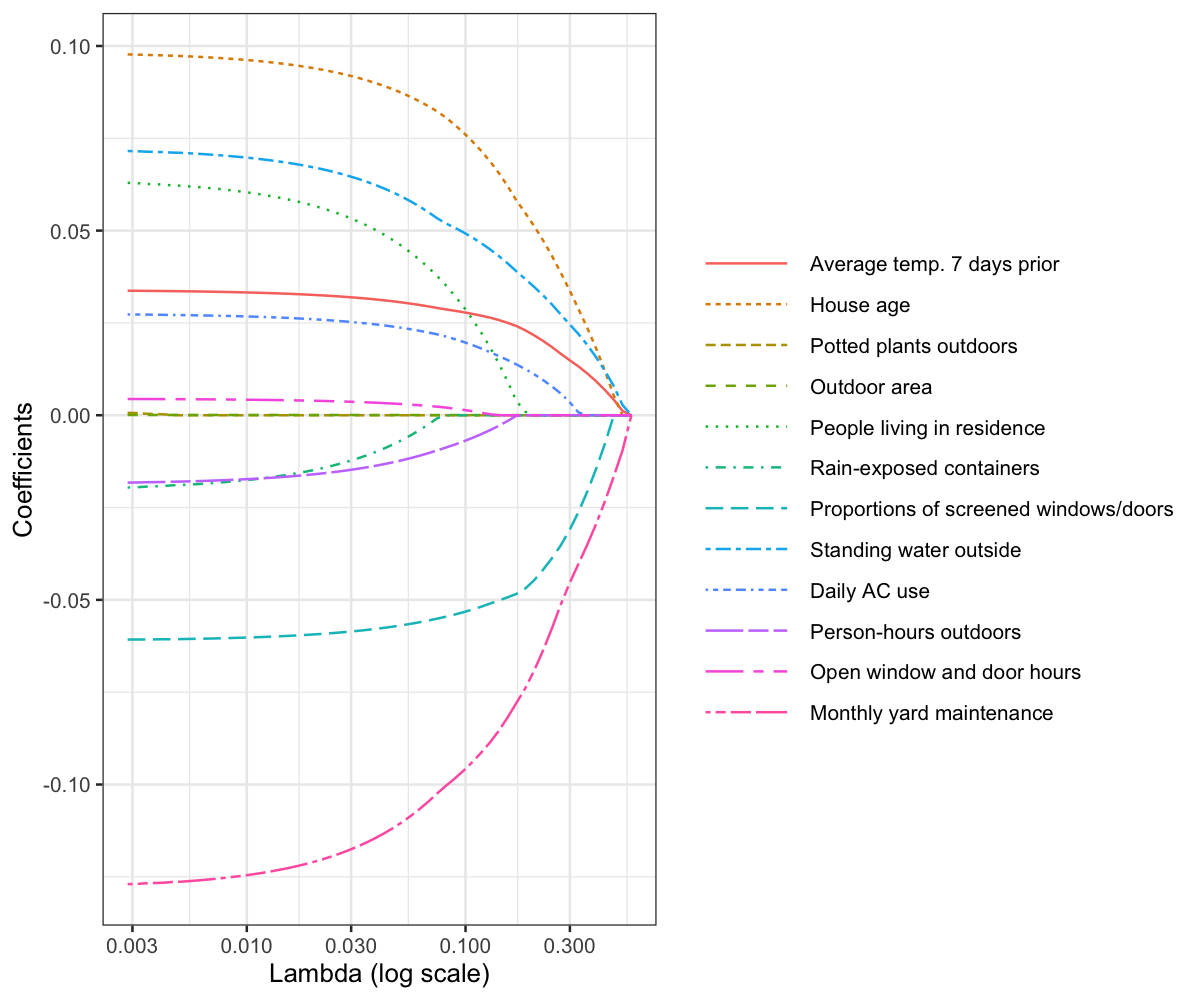


**Fig S3.** Standardized lasso coefficients for *Ae. aegypti* abundance measured outdoors. Monthly yard maintenance and standing water outside enter the model first, followed by house age, average temperature of 7 days prior, the proportion of screened windows and doors, and daily air-conditioner use. The last variables to enter the model are the number of people living in the residence, number of person-hours outdoors, number of open window and doors hours, number of rain-exposed containers, and number of potted plants outdoors.
