# Supplementary material for: Quantifying sociodemographic heterogeneities in the distribution of Aedes aegypti among California households
Source: PLoS Negl Trop Dis. 2020 Jul 21;14(7):e0008408. doi: 10.1371/journal.pntd.0008408 (PMC7394445; doi:10.1371/journal.pntd.0008408)
Supplement: S1 Table — (DOCX) [file pntd.0008408.s004.docx]

| **City** | **Boyle Heights** | **Commerce** | **East Los Angeles** | **Downey** | **La Mirada** | **Whittier** |
| --- | --- | --- | --- | --- | --- | --- |
| **Mean census tract median household income (range)** | $29,238 (21,299-37,242) | $45,603 (43,571- 46,596) | $39,808 23,095- 78,529) | $68,229 (51,500- 77,030) | $85,419 (60,568- 89,826) | $82,586 (48,783-137,793) |
| **Mean census tract population density (per sq. mile) (range)** | 10176 (2,279, 18,876) | 2942 (1,260, 3,695) | 19220 (2,562, 25,962) | 9463 (4,926, 15,182) | 6559 (2,278, 10,895) | 6620 (848, 11,937) |
| **Month of first *Aedes aegypti* detection** | August 2015 | October 2014 | August 2015 | August 2016 | November 2015 | August 2016 |
| **Number of households**  **surveyed** | 4 | 32 | 57 | 22 | 10 | 36 |

**Table S1. Income and population summary statistics for cities surveyed in Los Angeles County, California.** Census-tract data were collected from the American Community Survey 5-year estimates (2011-2016).
