# Supplementary material for: Quantifying sociodemographic heterogeneities in the distribution of Aedes aegypti among California households
Source: PLoS Negl Trop Dis. 2020 Jul 21;14(7):e0008408. doi: 10.1371/journal.pntd.0008408 (PMC7394445; doi:10.1371/journal.pntd.0008408)
Supplement: S2 Table — Household data were collected during the household surveys (2017) and census-tract data were collected from the American Community Survey 5-year estimates (2011–2016). (DOCX) [file pntd.0008408.s005.docx]

**Table S2.** Household and census-tract level predictors of *Ae. aegypti* abundance indoors and outdoors used in Poisson and quasi-Poisson models. Household data were collected during the household surveys (2017) and census-tract data were collected from the American Community Survey 5-year estimates (2011-2016).
