# Supplementary material for: Quantifying sociodemographic heterogeneities in the distribution of Aedes aegypti among California households
Source: PLoS Negl Trop Dis. 2020 Jul 21;14(7):e0008408. doi: 10.1371/journal.pntd.0008408 (PMC7394445; doi:10.1371/journal.pntd.0008408)
Supplement: S3 Table — The order of pairs corresponds to the order of pairs presented in Fig 2. (DOCX) [file pntd.0008408.s006.docx]

**Table S3.** Pearson’s correlation coefficients for all household and census tract level continuous variables among households in Los Angeles County surveyed for *Ae. aegypti* in 2017. The order of pairs corresponds to the order of pairs presented in Figure 2.
