# Supplementary material for: Quantifying sociodemographic heterogeneities in the distribution of Aedes aegypti among California households
Source: PLoS Negl Trop Dis. 2020 Jul 21;14(7):e0008408. doi: 10.1371/journal.pntd.0008408 (PMC7394445; doi:10.1371/journal.pntd.0008408)
Supplement: S4 Table — Models for Ae. aegypti counts indoors are Poisson regression models. Rate ratios and 95% confidence intervals are shown for all census-tract-level predictors included in the census-tract models. These models were adjusted for the mosquito collector, average daily temperature of the seven days prior to collection, and the collection date. (DOCX) [file pntd.0008408.s007.docx]

**Table S4.** Rate ratios for census-tract-level predictors of *Ae. aegypti* detections indoors. Models for *Ae. aegypti* counts indoors are Poisson regression models. Rate ratios and 95% confidence intervals are shown for all census-tract-level predictors included in the census-tract models. These models were adjusted for the mosquito collector, average daily temperature of the seven days prior to collection, and the collection date.
