# Supplementary material for: Quantifying sociodemographic heterogeneities in the distribution of Aedes aegypti among California households
Source: PLoS Negl Trop Dis. 2020 Jul 21;14(7):e0008408. doi: 10.1371/journal.pntd.0008408 (PMC7394445; doi:10.1371/journal.pntd.0008408)
Supplement: S5 Table — Models for outdoor Ae. aegypti counts were quasi-Poisson regression models. Rate ratios and 95% confidence intervals are shown for all census-tract-level predictors included in the census-tract models. These models were adjusted for the mosquito collector, average daily temperature of the seven days prior to collection, and the collection date. (DOCX) [file pntd.0008408.s008.docx]

**Table S5.** Rate ratios for census-tract-level predictors of *Ae. aegypti* detections outdoors. Models for outdoor *Ae. aegypti* counts were quasi-Poisson regression models. Rate ratios and 95% confidence intervals are shown for all census-tract-level predictors included in the census-tract models. These models were adjusted for the mosquito collector, average daily temperature of the seven days prior to collection, and the collection date.
