# Supplementary material for: Quantifying sociodemographic heterogeneities in the distribution of Aedes aegypti among California households
Source: PLoS Negl Trop Dis. 2020 Jul 21;14(7):e0008408. doi: 10.1371/journal.pntd.0008408 (PMC7394445; doi:10.1371/journal.pntd.0008408)
Supplement: S6 Table — Rate ratios and 95% confidence intervals are shown for all household predictors included in the model. This model was adjusted for the mosquito collector, average daily temperature of the seven days prior to collection, and the collection date. (DOCX) [file pntd.0008408.s009.docx]

**Table S6.** Rate ratios from the Poisson regression model for *Ae. aegypti* counts indoors. Rate ratios and 95% confidence intervals are shown for all household predictors included in the model. This model was adjusted for the mosquito collector, average daily temperature of the seven days prior to collection, and the collection date.
