# Supplementary material for: Quantifying sociodemographic heterogeneities in the distribution of Aedes aegypti among California households
Source: PLoS Negl Trop Dis. 2020 Jul 21;14(7):e0008408. doi: 10.1371/journal.pntd.0008408 (PMC7394445; doi:10.1371/journal.pntd.0008408)
Supplement: S7 Table — Rate ratios and 95% confidence intervals are shown for all household and census-level predictors included in the model. This model was adjusted for the mosquito collector, average daily temperature of the seven days prior to collection, and the collection date. (DOCX) [file pntd.0008408.s010.docx]

**Table S7.** Rate ratios from the hierarchical Poisson regression model for *Ae. aegypti* counts outdoors with a random variable for census tract. Rate ratios and 95% confidence intervals are shown for all household and census-level predictors included in the model. This model was adjusted for the mosquito collector, average daily temperature of the seven days prior to collection, and the collection date.
